# Time-restricted feeding and cognitive function in sedentary and physically active elderly individuals: Ramadan diurnal intermittent fasting as a model

**DOI:** 10.3389/fnut.2022.1041216

**Published:** 2022-11-09

**Authors:** Mohamed Ali Boujelbane, Khaled Trabelsi, Haitham A. Jahrami, Liwa Masmoudi, Achraf Ammar, Aïmen Khacharem, Omar Boukhris, Luca Puce, Sergio Garbarino, Egeria Scoditti, Saber Khanfir, Aymen Msaad, Amine Msaad, Soulaimane Akrout, Ahmed Hakim, Nicola Luigi Bragazzi, Kelsey Bryk, Jordan M. Glenn, Hamdi Chtourou

**Affiliations:** ^1^High Institute of Sport and Physical Education, University of Sfax, Sfax, Tunisia; ^2^Research Laboratory: Education, Motricité, Sport et Santé, EM2S, LR19JS01, High Institute of Sport and Physical Education of Sfax, University of Sfax, Sfax, Tunisia; ^3^Ministry of Health, Manama, Bahrain; ^4^College of Medicine and Medical Sciences, Arabian Gulf University, Manama, Bahrain; ^5^Department of Training and Movement Science, Institute of Sport Science, Johannes Gutenberg University Mainz, Mainz, Germany; ^6^Interdisciplinary Laboratory in Neurosciences, Physiology and Psychology, Physical Activity, Health and Learning (LINP2), UPL, UFR STAPS (Faculty of Sport Sciences), Paris Nanterre University, Nanterre, France; ^7^UVHC, DeVisu, Valenciennes, France; ^8^LIRTES-EA 7313, Université Paris Est Créteil Val De Marne, Créteil, France; ^9^Physical Activity, Sport, and Health, UR18JS01, National Observatory of Sport, Tunis, Tunisia; ^10^Department of Neuroscience, Rehabilitation, Ophthalmology, Genetics, Maternal and Child Health (DINOGMI), University of Genoa, Genoa, Italy; ^11^National Research Council (CNR)-Institute of Clinical Physiology (IFC), Lecce, Italy; ^12^Faculty of Medicine of Tunis, University of Tunis El Manar, Tunis, Tunisia; ^13^Laboratory of Pharmacology, Faculty of Medicine, University of Sfax, Sfax, Tunisia; ^14^Laboratory for Industrial and Applied Mathematics, Department of Mathematics and Statistics, York University, Toronto, ON, Canada; ^15^Neurotrack Technologies, Redwood City, CA, United States; ^16^Department of Health, Human Performance and Recreation, Exercise Science Research Center, University of Arkansas, Fayetteville, AR, United States

**Keywords:** religious fasting, exercise, time-restricted eating, elderly, cognitive performance, sleep quality

## Abstract

**Objectives:**

This study aimed to investigate the effects of Ramadan diurnal intermittent fasting (RDIF) on cognitive performance, sleep quality, daytime sleepiness, and insomnia in physically active and sedentary elderly individuals.

**Methods:**

A total of 58 participants (62.93 ± 3.99 years) were assigned to one of the following two groups: a sedentary group (control group) who observed Ramadan (*n* = 32) and a physically active group (*n* = 26) who continued to train while observing Ramadan. Participants were assessed 2 weeks before Ramadan and during the fourth week of Ramadan. On each occasion, participants completed a digital assessment of their cognitive performance and responded to the Pittsburgh sleep quality index (PSQI), the insomnia severity index (ISI) and the Epworth sleepiness scale (ESS) questionnaires to assess sleep parameters.

**Results:**

Compared to before Ramadan, performance in executive function (*p* = 0.035), attention (*p* = 0.005), inhibition (*p* = 0.02), associative memory (*p* = 0.041), and recognition memory (*p* = 0.025) increased significantly during Ramadan in the physically active group. For the sedentary group, associative learning performance decreased (*p* = 0.041), whilst performances in the remaining domains remained unchanged during Ramadan. Global PSQI, ISI, and ESS scores indicated both groups suffered from poor sleep quality and excessive daytime sleepiness, with significantly higher negative effects of RDIF observed in the sedentary group.

**Conclusion:**

Older adults who continue to train at least three times per week during Ramadan may improve their cognitive performance, despite the impairment of sleep quality. Future studies in older adults during Ramadan including objective measures of sleep (e.g., polysomnography, actigraphy) and brain function (e.g., functional magnetic resonance imaging) are warranted.

## Introduction

The incidence and severity of brain disease is steadily rising over the past decades and one of the leading causes of disability and death worldwide (1, 2).

Age-related cognitive decline is inextricably linked to the highly complicated biological process of brain aging (3), which is the primary risk factor for neurodegeneration (4). This brain health deterioration is characterized by neural loss, reduced neurogenesis, and limited neuronal plasticity (5), which are associated with subsequent declines in memory, executive function, attentional abilities, and processing speed (6, 7). Additionally, physiological alterations due to the aging process contribute to disturbances in the circadian rhythms (i.e., central and peripheral rhythms) (8), including a decrease in overall melatonin secretion (9–11), as well as metabolism-related rhythms (e.g., lipid metabolism, glucose control, xenobiotic detoxification), regulated by the liver and pancreatic clocks which can lead to the emergence of metabolic diseases (12).

Diet and the gut-brain axis have recently been recognized for their contribution to brain health and cognitive function (13, 14), thus the ability for dietary treatments to prevent and/or treat brain diseases is promising. As such, intermittent fasting which involves abstaining from meals or severely restricting caloric intake for durations ranging from 12 to 48 h, has demonstrated potential effects on brain health through the enhanced expression of brain-derived neurotrophic factor (BDNF), enhancing knowledge acquisition and retention, stimulating neurogenesis, and consequently, improving cognitive function (15–21).

Ramadan diurnal intermittent fasting (RDIF) is considered a unique model of intermittent fasting (i.e., religious and spiritual reasons) in which Muslims abstain from eating, drinking, smoking and sexual intercourses between dawn and sunset for a lunar month (i.e., 28–30 days) every year (22). In this context, Alsharidah et al. (23) demonstrated the beneficial effects of RDIF in adults on psychomotor function, attention, and processing speed. Likewise, Farooq et al. (24) showed a significant increase in performance for spatial planning, working memory tasks, and working memory capacity tests, at the end of Ramadan month. However, Ghayour Najafabadi et al. (25) showed RDIF has negative effects on short-term memory and cognitive flexibility function.

Several influencing factors can affect cognitive performance, such as disruption of sleep patterns, meal composition and schedule, and lifestyle changes (26–28).

Previous studies showed impairment of sleep quantity (i.e., nocturnal sleep duration) and quality and excessive daytime sleepiness (EDS) during Ramadan, possibly due to physiological and behavioral changes in diurnal and nocturnal activities. These activities include waking up for the predawn meal (*Suhur*) and dawn prayer, and practicing more activities at night such as social meetings, shopping, *Quran* reading groups, and prayers (e.g., *Attarawih*) (29–31).

Physical activity (PA) may attenuate some of the negative impacts that poor sleep has on cognition and improve cognitive function through physiological adaptations such as neurogenesis, angiogenesis, increases in cerebral blood flow, functional connectivity, and growth factors, while reducing inflammation and brain neuropathology accumulation (32–36). Furthermore, Küster et al. (37) concluded that a regular active lifestyle is associated with better global cognition in elderly individuals. However, during the month of Ramadan, Farooq et al. (38) showed a decrease in the objectively assessed habitual PA in adults. Similarly, Lessan et al. (39) reported that the total number of steps walked per day were significantly lower during Ramadan.

Some investigations have examined the effect of RDIF on cognitive function and sleep quality in adolescents (24, 40, 41) and athletes (42, 43), but not in physically active and sedentary older adults. Examining the effects of combining regular physical exercise and intermittent fasting in older adults may enable us to assess whether combining intermittent fasting and regular training is an effective therapeutic strategy for the prevention or even the improvement of cognitive function, thus potentially counteracting the adverse effects associated with the aging process.

Therefore, our primary objective is to investigate the effect of RDIF on cognitive function in active and sedentary older adults. Our secondary objective is to examine the influence of RDIF on sleep quality, daytime sleepiness, insomnia, and sleep-wake behaviors in active and sedentary older adults.

We hypothesized that from before to during RDIF, (i) active older adults would have better cognitive performance than their sedentary counterparts, and (ii) both groups would have a sleep quality impairment, higher sleepiness and insomnia more accentuated in the sedentary group.

## Materials and methods

### Participants

**FIGURE 1:**
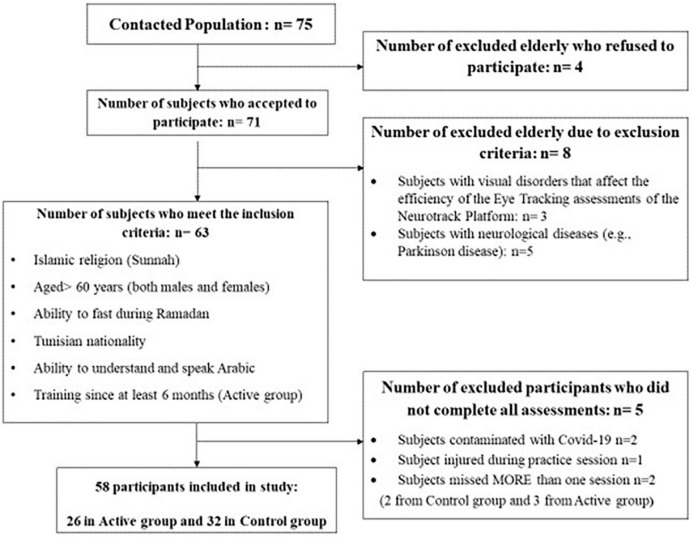
Flowchart outlining the selection criteria and decision-making process for the elderly who participated in the ongoing assessments.

Seventy-one older adults (aged > 60 years) agreed to participate in this study. After the verification of eligibility criteria, 63 subjects signed an informed consent prior to the start of the evaluation process (Figure 1). Five participants were excluded because they did not complete all assessments. A total of 58 included participants (26 men and 32 women) aged between 60 and 79 years (62.9 ± 4.0 years) were assigned to one of the following two groups: Control group (*n* = 32; mean age: 62.71 ± 3.52 years; age range: 60–77) who did not follow any training program before Ramadan and physically active elderly group (active group) (*n* = 26; mean age: 63.19 ± 4.56 years; age range: 60–78) who (i) trained for at least 6 months prior to RDIF. The active group continued with the same training schedule while observing Ramadan (Table 1), and completed all tests at the final analysis. Participant characteristics are presented in Table 2. There were some participants with chronic and/or cardiovascular disease and, based on medical doctors’ authorization, their participation in the training program and/or the fasting during Ramadan does not pose any risk of danger to their health. The rate of compliance with the training program was 93.59%. The research ethics committee CPP SUD N°0322/2021 gave its approval to this project, and the present study was carried out according to the Declaration of Helsinki. The required sample size was calculated *a priori* for repeated measures analysis of variance (ANOVA) using the G* power software (version 3.1.9.2; Kiel University, Kiel, Germany) (44). Values for α were set at 0.05 and power at 0.90. Given the pioneer character of this study and since no previous study has evaluated the effects of RDIF on cognitive function in older adults, an effect size *f* of 0.4 was reported in the study of Tian et al. (42) for the verbal learning and memory assessed at16:00 h during Ramadan. A total sample size of 52 was required with at least 26 in each group to achieve the desired power for cognitive functions parameters. Concerning sleep components, based on the results of the meta-analysis of Trabelsi et al. (31), an effect size *f* of 0.41 was computed for the nocturnal sleep duration. The required sample size was 25 for each group for nocturnal sleep duration. To avoid potential risks of participant drop-out, a total of 58 participants were included.

**TABLE 1:** Training program for active group before and during Ramadan.

3 sessions per week of 1 h interspersed with a recovery day		
Session 1	Session 2	Session 3
• 15 min warm-up	• 15 min warm-up in the pool	• 15 min warm-up
• 35 min team sports (i.e., volleyball or basketball)	• 35 min aquatic activities	• 35 min circuit training and ludic games.
• 10 min stretching	• 10 min stretching in the pool	• 10 min stretching

**TABLE 2:** Demographic baseline characteristics of the participants.

	Variables	Active group (*n* = 26)	Control group (*n* = 32)
Mean (*SD*)	BMI	26.6 (2.8)	27.3 (2.7)
***N* (%)**	**Gender**		
	Female	13 (50%)	18 (56.3%)
	Male	13 (50%)	14 (43.7%)
	**Education level**		
	University	11 (42.3%)	7 (21.9%)
	High school	9 (34.6%)	8 (25%)
	Mild school	5 (19.2%)	12 (37.5%)
	Primary school	1 (3.8%)	5 (15.6%)
	**Health status**		
	Healthy	14 (53.8%)	19 (59.4%)
	**Chronic diseases**		
	Type 2 diabetes	5 (19.2%)	6 (18.8%)
	Dyslipidemia	1 (3.8%)	1 (3.1%)
	**Cardiovascular diseases**		
	Coronary artery disease	0 (0%)	1 (3.1%)
	Hypertension	3 (11.5%)	2 (6.3%)
	Type 2 diabetes + hypertension	1 (3.8%)	2 (6.3%)
	Type 2 diabetes + dyslipidemia	2 (7.7%)	1 (3.1%)

BMI, Body mass index; SD, Standard deviation.

### Experimental design

The study was carried out in Tunisia during the 2022 Ramadan month from 02 April to 02 May in the Spring, when daily fasting started from ∼04:35 a.m. and ended at ∼6:45 p.m. (i.e., fasting duration of approximately ∼14 h). Participants were assessed twice: 2 weeks prior to Ramadan (non-fasting condition), in which the mean temperature was 15 ± 2.1n°C and the mean humidity 76 ± 10.2%, and during the fourth week of Ramadan’s fast (fasting condition) with 18 ± 1.0°C of mean temperature and 72 ± 8.3% of mean humidity.

All tests were conducted at the same time of day between 4:00 and 6:00 p.m. for both groups before and during RDIF (before the break of fast). Participants had their last meal between 03:30 and 04:30 a.m. (i.e., *Suhur*), approximately 11–12 h before their assessment session.

The two sessions were conducted in a laboratory under the guidance of a qualified examiner who directed the evaluation process and provided subjects with instructions, cues, and information as needed. During Ramadan, participants (active group) continued to train in a fasted state (i.e., at the same time as before Ramadan (i.e., 5:30 p.m.) while the control group did not perform any type of physical training program. All subjects fasted during the entire month of Ramadan. No side effects (i.e., dizziness, hypoglycemia, headache) due to fasting were recorded in either group, suggesting the feasibility of the intervention.

Both groups performed the same evaluation procedure; each session included a digital cognitive assessment battery (Neurotrack Technologies, Redwood City, CA), an assessment of sleep, sleepiness and insomnia based on validated Arabic version questionnaires including the Pittsburgh sleep quality index (PSQI) (45, 46), the ESS (47, 48) and the insomnia severity index (ISI) (49, 50).

### Cognitive assessments

Cognitive function was assessed using the Neurotrack digital cognitive battery. Neurotrack’s cognitive assessments are brief, reliable measures that are based on paradigms previously shown to be sensitive to fluctuations in cognitive function (51–53). Given the assessments are designed in American English, the battery was adapted to fit our sample population.

First, the assessments and instructions were converted into an explanatory video with an Arabic language voice. Second, the output responses on the keyboard buttons were colored and translated to Arabic (e.g., yes, no, left, right, equal, unequal). The translation-back translation was carried out by two native Arabic speakers who were also fluent in English. To ensure understanding and feasibility, the translated versions of the assessments were pilot-tested with 15 older adults who were not included in the final sample. The pilot feedback was incorporated into the translated assessments, which were then re-piloted until no additional changes were required based on subsequent feedback. This digital cognitive battery was performed in translated Arabic version as in the recent study conducted by Boujelbane et al. (54).

This digital cognitive battery consists of six tests, each with its own measurement domain:

– Symbol Match, a digitized version of the traditional Digit Symbol Substitution test, is a task that assesses processing speed and executive functioning. During this test, scores are calculated based on speed and accuracy.
– Item Price is a visual associative learning measure. Participants in a recognition trial are required to learn a succession of food/price pairs and then identify appropriately matched pairs. All of the items fall into the same semantic category (e.g., vegetables, and fruits). The accuracy of the answers determines the score.
– Arrow Match, based on the traditional Choice Reaction Time paradigm, is a test of attention and processing speed. Five arrows appear in the center of the screen, and participants are asked to identify/determine the direction of the middle arrow. Speed and accuracy are used to calculate scores.
– Path Points is an executive function and attention measure developed based on the Trail Making Test-Part B. Participants are instructed to alternately connect a succession of circles with numbers and letters (e.g., 1 connected to A, 2 connected to B, 3 connected to C, etc.). Speed determines the score.
– Light Reaction is an inhibition measure based on the go/no-go paradigm. A positive (i.e., green light) or negative (i.e., red light) stimulus will be shown to participants. In the case of a positive stimulus appearance, the participant must press a button. However, in the case of the appearance of a negative stimulus appears, subjects are instructed not to press the button. Speed and accuracy are used to calculate scores.
– Image Pairs is a test that assesses associative and recognition memory. The latter is assessed *via* eye-tracking movements (ETM) (55). Behavioral responses are used to assess the associative memory component of the assessment. Briefly, a series of object pairs are presented to subjects during the familiarization phase. Then, during the test phase, subjects are asked to identify the previously observed pairs. Participants are instructed to look at and focus just on the new (i.e., novel) image that has not previously been shown. The individual’s “novelty preference” is determined by the proportion of time spent viewing the new image relative to the overall gazing time. Accuracy is used to calculate scores.

### The Epworth sleepiness scale

The subjective sleepiness was assessed using the Arabic validated version of the ESS questionnaire (56). On a four-point scale, participants indicated how prone they are to doze in eight different daily situations (circumstances). ESS scores were interpreted as follows—0–5: lower than normal daytime sleepiness; 6–10: higher than normal daytime sleepiness; 11–12: mild excessive daytime sleepiness (EDS); 13–15: moderate EDS and 16–24: severe EDS (57). In older men, the internal consistency is adequate (Cronbach’s α = 0.70) (58). For the construct validity, worse sleep on the PSQI is associated with greater sleepiness on the ESS (*r* = 0.13; *p* < 0.001) (58).

### The Pittsburg sleep quality index

The PSQI questionnaire (45), extensively validated in various populations and cultures (59), was used to assess sleep quality over the preceding month. The Arabic-validated version of the PSQI was utilized (46). The questionnaire includes 19 questions, each representing one of the seven sleep quality components: subjective sleep quality, sleep duration, sleep onset latency (SOL), sleep disturbance, sleep efficiency, daytime dysfunction, and sleep medication intake (45). Global (total) PSQI scores > 5 indicate poor sleep quality, whilst global PSQI scores ≤ 5 indicate good sleep quality (45). The reliability of the test according to Cronbach’s α is 0.81 (60). In addition, the internal consistency of the PSQI was 0.81 and the scales correlation score ranged from 0.48 to 0.71 (60).

### Insomnia severity index

The Arabic-validated version of the ISI questionnaire was utilized (50). The ISI is a self-reported 7-item questionnaire that assesses the nature, severity, and impact of insomnia. ISI scores range from 0 to 28. The total ISI scores of 8 or higher indicates insomnia. The total score is interpreted as follows: no insomnia (0–7); sub-threshold insomnia (8–14); moderate insomnia (15–21); and severe insomnia (22–28). The internal consistency reliability equals 0.91 (49, 61). Regarding validity, ISI shows a statistically significant positive correlation with Athens Insomnia scale-5 (*r* = 0.93) (49, 61).

### The Arabic version of the physical activity scale for the elderly

The PASE is a brief, easy-to-score, reliable, and valid instrument used in epidemiologic investigations to assess PA in the elderly (62). Respondents are asked to report the number of days per week in which the activity was performed, as well as the number of hours per day spent on leisure activity items. PASE scores are calculated from weights and frequency values assigned to each of the 12 types of activity. The internal consistency of the physical activity scale for the elderly (PASE-A) components is good (Cronbach’s α 0.70–0.75), and the reliability of the components is excellent (ICC2,10.90–0.98) (63).

### Statistical analysis

All statistical analyses were performed using STATISTICA software (StatSoft, version 12, Paris, France). Data are presented as means and standard deviations. The variation (Δ) recorded from before Ramadan session to during Ramadan session is calculated as follows: Δ = During Ramadan – Before Ramadan. The percentage of variation (Δ%) recorded from before Ramadan session to during Ramadan session is calculated by the following formula: Δ% = (Δ/Before Ramadan) × 100. The normality of the distributions was tested by the Shapiro-Wilks test.

Pearson’s chi-squared test was used to compare the demographic baseline characteristics of the participants including gender, education level, and health status of the two groups. Because the age and the body mass index (BMI) were not normally distributed, the non-parametric Mann-Whitney *U*-test was used.

From all investigated parameters, processing speed, associative learning, executive function, attention, and ESS scores are normally distributed. Therefore, a two-way mixed analysis of variance (ANOVA) was applied 2 groups (Sedentary vs. Active) × 2 Ramadan (Before Ramadan vs. During Ramadan) for these variables. When the ANOVA showed a significant interaction, pairwise comparison is carried out by a Bonferroni *post-hoc* test, otherwise by an independent or paired Student’s *t*-test.

For other parameters where normality is not verified, non-parametric tests are used as follows: Wilcoxon test was made for the comparison from before to during Ramadan for each group and the Mann-Whitney *U*-test is carried out for the comparison between the means of the two groups, before Ramadan, during Ramadan and the Δ variation. All statistics are considered significant for a probability threshold of 5% (*p* < 0.05).

To determine the meaningfulness of significant findings for the ANOVA Group × Ramadan interaction, effect sizes (ES) were calculated as partial eta-squared η*_*p*_*^2^ (64, 65). Small, moderate, and large ES are represented by η*_*p*_*^2^ values of 0.01, 0.06, and 0.14, respectively (65). Furthermore, the magnitude of the change in score was determined using Cohen’s *d* (66), and it was interpreted using the following criteria: 0.2 (small), 0.5 (moderate), and 0.8 (large). The matched rank biserial correlation was used to measure ES for non-parametric tests (67). ESs were classified as small (0.10–0.30), medium (0.30–0.50), and large (0.50) (67).

## Results

### Baseline characteristics of participants

There were no significant differences between active and sedentary participants with regard to gender, education level, and health status. Likewise, the Mann-Whitney *U*-test reported no significant difference in age and BMI between groups.

### Cognitive function

**FIGURE 2:**
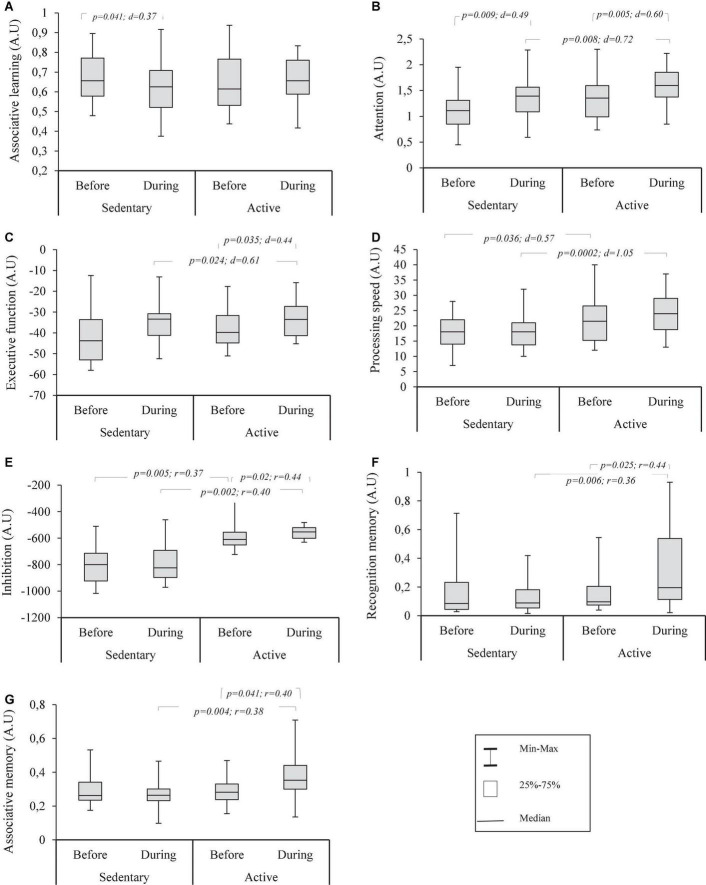
Box plots distribution of recognition memory **(A)**, associative memory **(B)**, inhibition **(C)**, processing speed **(D)**, associative learning **(E)**, executive function **(F)** and attention **(G)** assessed using Neurotrack’s digital cognitive battery in sedentary and active groups before and during Ramadan fasting.

Cognitive function before and during Ramadan are shown in Figure 2. The two-way ANOVA (Ramadan × group) for associative learning (i.e., Figure 2A) showed no significant effect for Ramadan × group interaction [*F*_(1,56)_ = 2.12; *p* = 0.151; η*_*p*_*^2^ = 0.04]. Paired samples *t*-test revealed that associative learning decreased significantly in the control group with no change in the active group (*p* = 0.738; *d* = 0.066). Independent samples *t*-test showed no significant differences in this variable between the two groups at any time period.

For attention (i.e., Figure 2B), there was no significant Ramadan × group interaction [*F*_(1,56)_ = 0.31; *p* = 0.581; η*_*p*_*^2^ = 0.005]. From before to during RDIF, a significant increase in both groups was shown with greater scores for the active group during RDIF. Before RDIF, no significant difference between the two groups was recorded (*p* = 0.056; *d* = 0.516).

Executive function (i.e., Figure 2C) revealed no significant effect for Ramadan × group interaction [*F*_(1,56)_ = 2.77; *p* = 0.102; η*_*p*_*^2^ = 0.047]. However, a significant increase from before to during RDIF was found in the active group, whereas this variable remained unchanged in the control group during the same time period (*p* = 0.584; *d* = 0.098). Moreover, no significant difference between the groups existed before RDIF (*p* = 0.933; *d* = 0.022), while better scores were observed in the active group during RDIF.

Likewise, there was no significant effect for Ramadan × group interaction [*F*_(1,56)_ = 1.98; *p* = 0.165; η*_*p*_*^2^ = 0.034] on processing speed (i.e., Figure 2D). No change from before to during RDIF either for active (*p* = 0.101; *d* = 0.334) and control groups (*p* = 0.701; *d* = 0.068). Furthermore, a significant difference between the two groups before and during RDIF was observed.

With regard to inhibitory function (i.e., Figure 2E), the Mann–Whitney *U*-test revealed no significant effect for Ramadan × group interaction (*z* = 0.899; *p* = 0.369; *r* = 0.118). The Wilcoxon test showed inhibitory function increased significantly in the active group while there was no change from before to during RDIF for the control group (*p* = 0.614; *r* = 0.089). Compared to the sedentary group, higher scores were recorded for the active group before and during RDIF.

For recognition memory (i.e., Figure 2F), a significant effect for Ramadan × group interaction (*z* = 2.291; *p* = 0.022; *r* = 0.301) was shown. The Bonferroni *post-hoc* test revealed a significant increase from before to during RDIF for the active group; this parameter remained unchanged for the control group (*p* = 0.466; *r* = 0.129) throughout the study. No difference between groups was found before RDIF (*p* = 0.477; *r* = 0.093), whereas higher scores were recorded for the active group during RDIF compared to the control group.

For associative memory (i.e., Figure 2G), there was a significant effect for Ramadan × group interaction (*z* = 2.556; *p* = 0.011; *r* = 0.336). The Bonferroni *post hoc* test revealed a significant increase throughout the investigation for the active group, while no change was observed for the sedentary group (*p* = 0.130; *r* = 0.268). Furthermore, no difference between groups was shown before RDIF (*p* = 0.969; *r* = 0.005), whilst the active group had a greater score during RDIF than the control group.

**FIGURE 3:**
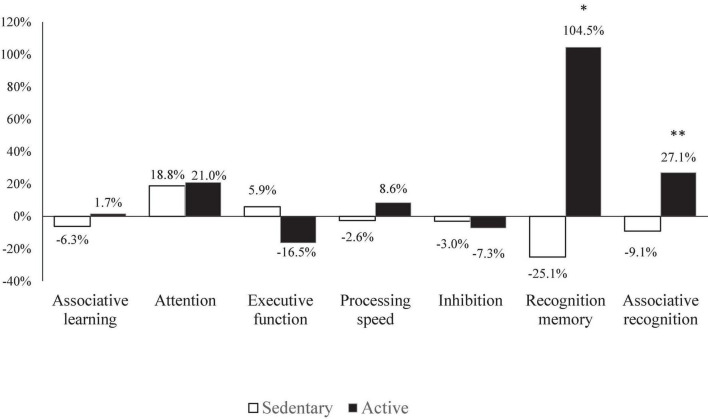
Percent change (Δ%) of cognitive functions for sedentary and active groups. *Significant difference: *p* < 0.05; **significant difference: *p* < 0.001.

Figure 3 shows the percent change (Δ%) of cognitive function from before to during RDIF in both groups. There was a significant effect for Ramadan × group interaction between sedentary and active groups in the memory parameters (i.e., recognition memory, associative recognition), with a higher positive Δ% observed in the active group.

### Effects of Ramadan on sleep quality quantitative sleep measures, daytime sleepiness, insomnia, and physical activity level

The means scores of quantitative measures of PSQI questionnaires, daytime sleepiness, insomnia, and PA level before and during Ramadan are presented in Table 3.

**TABLE 3:** Means and standards deviations (SD) of questionnaire findings before and during Ramadan for sedentary and active groups.

Parameters	Groups	Means ± *SD*				Ramadan × group interaction		
			
		Before-R	During-R	Delta	Δ %	F/Z	*P*-value	η*_*p*_*^2^/*r*
ESS score (a.u.)	Sedentary	6.41 ± 2.96	7.69 ± 3.12a	1.28 ± 2.22	20.00%	*F* = 0.030	0.859	η_*p*_^2^ = 0.001
	Active	4.46 ± 1.79b	5.65 ± 2.28ab	1.19 ± 1.36	26.70%			
ISI score (a.u.)	Sedentary	5.91 ± 2.9	6.84 ± 3.61a	0.94 ± 2	15.90%	*z* = 0.133	0.894	*r* = 0.017
	Active	3.58 ± 2.8b	4.46 ± 2.89ab	0.88 ± 1.88	24.70%			
Global PSQI score (a.u.)	Sedentary	6.38 ± 2.15	9.69 ± 1.96a	3.31 ± 1.69	52.00%	*z* = 2.494	0.013	*r* = 0.327
	Active	5.81 ± 2.17	8.04 ± 2.34ab	2.23 ± 2.14	38.40%			
Subjective sleep quality (a.u.)	Sedentary	0.88 ± 0.79	1.66 ± 0.94a	0.78 ± 1.01	89.30%	*z* = 1.243	0.214	*r* = 0.163
	Active	0.54 ± 0.71	1.12 ± 1.11ab	0.58 ± 0.9	107.10%			
Sleep disturbances (a.u.)	Sedentary	0.53 ± 0.51	1.22 ± 0.55a	0.69 ± 0.59	129.40%	*z* = 0.571	0.568	*r* = 0.075
	Active	0.31 ± 0.47	0.85 ± 0.46ab	0.54 ± 0.71	175.00%			
Daytime dysfunction (a.u.)	Sedentary	0.47 ± 0.67	1.34 ± 0.65a	0.88 ± 0.71	186.70%	*z* = 1.415	0.157	*r* = 0.186
	Active	0.12 ± 0.33	0.77 ± 0.51ab	0.65 ± 0.49	566.70%			
Sleep latency (min)	Sedentary	18 ± 11.6	25.8 ± 13.6a	7.78 ± 8.64	43.20%	*z* = 1.767	0.077	*r* = 0.232
	Active	19 ± 12.6	23.1 ± 11a	4.12 ± 7.48	21.70%			
Sleep efficiency (%)	Sedentary	89.4 ± 8.2	84.2 ± 9.3a	–5.28 ± 7.22	–5.90%	*z* = 1.681	0.093	*r* = 0.221
	Active	85.7 ± 10	84.3 ± 7.5	–1.43 ± 9.38	–1.70%			
Nocturnal sleep duration (hours)	Sedentary	7.22 ± 1.11	6.17 ± 0.96a	–1.05 ± 1.09	–14.50%	*z* = 2.853	0.004	*r* = 0.375
	Active	7.02 ± 1.07	6.73 ± 1.03b	–0.29 ± 0.75	–4.10%			
Bedtime (h:mm)	Sedentary	23:30 ± 1:07	00:39 ± 1:22a	1:09 ± 1:04	4.9%	*F* = 5.39	0.024	η*_*p*_*^2^ = 0.088
	Active	23:06 ± 1:05	23:40 ± 0:53ab	0:34 ± 0:48	2.4%			
Wake-up (h:mm)	Sedentary	6:43 ± 1:07	6:49 ± 1:20	0:06 ± 1:00	1.5%	*F* = 0.79	0.377	η*_*p*_*^2^ = 0.014
	Active	6:07 ± 1:04b	6:25 ± 0:57a	0:18 ± 0:33	4.8%			
Use of sleep medication (a.u.)	Sedentary	0.09 ± 0.3	0.06 ± 0.25	–0.03 ± 0.31	–33.30%	*z* = 0.031	0.975	*r* = 0.004
	Active	0.08 ± 0.39	0.04 ± 0.2	–0.04 ± 0.2	–50.00%			
PASE score (a.u.)	Sedentary	101.23 ± 20.6	84.383 ± 13.11a	–16.84 ± 12.52	–20%	*z* = 2.447	0.014	*r* = 0.321
	Active	153.81 ± 11.14b	119.55 ± 11.25ab	–34.26 ± 16.22	–28.70%			

a: significantly different from before Ramadan at *p* < 0.05; b: significantly different from sedentary group at *p* < 0.05. Before-R, before Ramadan; During-R, during Ramadan; PSQI, Pittsburgh Sleep Quality Index; ESS, Epworth sleepiness scale; ISI, insomnia severity index; PASE, Physical activity scale for elderly; Δ, difference between during and before Ramadan; *SD*, standard deviation; a.u., arbitrary unit.

Before RDIF, there were significant differences between the two groups in ESS (*p* = 0.003; *d* = 0.578), ISI (*p* = 0.013; *r* = 0.440) and PASE (*p* < 0.001; *r* = 0.853) scores, in which control group had worst scores compared to active group.

From before to during RDIF, ESS score, ISI score, Global PSQI score, subjective sleep quality, sleep disturbances, daytime dysfunction increased in active group (*p* < 0.001; *d* = 0.879, *p* = 0.031; *r* = 0.422, *p* < 0.001; *r* = 0.781, *p* = 0.007; *r* = 0.531, *p* = 0.005; *r* = 0.552, *p* < 0.001; *r* = 0.710, respectively) and control group (*p* = 0.003; *d* = 0.578, *p* = 0.013; *r* = 0.440, *p* < 0.001; *r* = 0.852, *p* = 0.001; *r* = 0.580, *p* < 0.001; *r* = 0.683, *p* < 0.001; *r* = 0.717, respectively), with significantly higher negative effects observed during RDIF in control group (*p* = 0.007; *d* = 0.733, *p* = 0.016; *r* = 0.315, *p* = 0.006; *r* = 0.361, *p* = 0.043; *r* = 0.266, *p* = 0.038; *r* = 0.273, *p* = 0.004; *r* = 0.380, respectively).

Furthermore, sleep latency increased in active (*p* = 0.025; *r* = 0.439) and sedentary (*p* < 0.001; *r* = 0.672) groups, with no significant differences between the two groups at any time periods. Moreover, there was a significant increase in habitual sleep efficiency only for the control group (*p* < 0.001; *r* = 0.718), while for the active group this parameter remained unchanged (*p* = 0.411; *r* = 0.161).

Sleep duration decreased significantly from before to during RDIF in the control group (*p* < 0.001; *r* = 0.770), while no significant change observed in the active group (*p* = 0.057; *r* = 0.373). The sedentary population had lower sleep duration during RDIF (*p* = 0.036; *r* = 0.275).

Bedtime was delayed by 0.56 h during vs. before Ramadan in active group (*p* = 0.002, *d* = 0.7). Likewise, the inactive group showed a delay in bedtime by 1.15 h from before to during Ramadan (*p* < 0.001, *d* = 1.1). A significant difference was found between the two groups during Ramadan (*p* = 0.003, *d* = 0.8). However, no significant difference between active and sedentary groups was shown before Ramadan.

Active group awakened 0.3 h later during vs. before Ramadan (*p* = 0.01, *d* = 0.5), whereas no significant difference was found in the sedentary group (*p* = 0.580, *d* = 0.1). No significant difference was found between the two groups during Ramadan.

No significant change and/or difference was recorded in both groups for the use of sleep medication (*p* > 0.05).

Finally, PASE total score decreased significantly in active (*p* < 0.001; *r* = 0.874) and sedentary (*p* < 0.001; *r* = 0.803) groups from pre to during RDIF, with preservation of greater values during RDIF in active group (*p* < 0.001; *r* = 0.827).

## Discussion

The present study is the first to evaluate the effects of RDIF on cognitive function, sleep quality, daytime sleepiness, insomnia, and sleep-wake behaviors for active and inactive elderly populations.

Before RDIF, significant differences in questionnaires baseline scores including ESS, ISI, and PASE as well as for some cognitive function domains (i.e., inhibition and processing speed) were reported. This may be explained by the physical training effects in the active group. Previous reports suggest a beneficial role of chronic training schedule on inhibitory function (68, 69), processing speed (70), insomnia, daytime sleepiness, and sleep quality (71, 72).

Furthermore, from before to during RDIF, our results showed an improvement in the majority of cognitive function variables for the active group, including attention, executive function, inhibition, recognition memory, and associative memory; there were no changes for other cognitive domains including processing speed and associative learning. However, the control group showed a significant decline in associative learning from before to during RDIF, unlike other cognitive domains which remained unchanged overtime period. These findings partially verified our first hypothesis. Furthermore, we observed an overall deterioration of sleep quality and an increase in daytime sleepiness for both groups. The reported scores of PSQI and ESS confirmed both groups suffered from poor sleep quality, which confirmed our second hypothesis.

The average overall PSQI score (PSQI score ≥ 5) recorded before and during Ramadan is indicative of poor sleep quality (43, 73), which could be associated with volume loss in several hippocampal subfields triggered by the aging process (74). The greater increase in the global PSQI score during RDIF (PSQI score > 8) (75) for the control group suggests a worsened sleep quality and thus potential depressive syndrome (76). Based on the results of a recent systematic review (77), it is likely that maintaining a moderate intensity PA during Ramadan could have reduced the negative effect of Ramadan on sleep quality. Furthermore, several studies have shown exercise may modulate the circadian rhythm by serving as a time cue, modifying the phase of a molecular clock in peripheral tissues (78–80). More precisely, endurance and resistance exercise may stimulate the expression of core molecular clock genes, through the influence on exercise-sensitive genes such as AMPK, HIF-1α, and PGC1α. Increased activation of AMPK modifies the stability of Per and Cry, modulating the expression of clock genes (78–81). Moreover, adopting PA can potentially resynchronize peripheral muscle and vascular clock misalignment, which could improve vascular health and, consequently, skeletal muscle function (82).

Regarding PSQI components, nocturnal sleep duration, which is a critical element in assessing the restorative effects of sleep (83), decreased more in the control group (<7 h/night) by ∼1 h during RDIF, as older adults require about the same amount of sleep as all adults (i.e., 7–9 h/night) (84). The decrease in nocturnal sleep duration was previously reported during Ramadan (30, 85) and could be explained by the occurrence of several lifestyle changes in nocturnal and diurnal activities (i.e., zeitgeber factors), such as rising early for the *Suhur* meal and engaging in more social activities after the break of fast (e.g., *Quran* reading groups, social gatherings, shopping, prayers) (30, 86, 87). Furthermore, the greater than normal food consumption at night as well as eating close to bedtime could have decreased nocturnal sleep duration by negatively affecting circadian rhythm and hormone excretion (88, 89).

Sleep disturbances increased significantly in both groups during RDIF. Nocturnal urination is known as a potential cause of sleep disturbances in the elderly (90), and increased water intake after the break of fast during RDIF could have exacerbated the effect of nocturnal urination on sleep disturbances. Additionally, rising early for the *Suhur* and prayers could explain, at least in part, the previous finding.

SOL, another component of PSQI, increased in both groups. Additionally, average SOL values reported during Ramadan in the two groups were higher than the cut-off of 20 min indicative of usual SOL (91), suggesting our participants have difficulty initiating sleep during Ramadan. The increased exposure to evening light during Ramadan (e.g., family commitment, watching TV programs, shopping) could have prolonged subsequent SOL (i.e., taking longer to fall asleep during Ramadan) in both groups. As a result of sleep impairment, daytime dysfunction increased during Ramadan in the two groups; this may increase the loss of daily structure during Ramadan (92).

ESS scores increased during Ramadan in the two groups. However, it is worth mentioning that the average ESS score recorded in the sedentary group is indicative of higher than normal daytime sleepiness, whilst daytime sleepiness in the active group is lower than normal. The increase in daytime sleepiness could be explained by sleep insufficiency during Ramadan, as previously reported by Faris et al. (30). In this context, a comparable increase in daytime sleepiness levels during Ramadan was previously reported by the systematic review and meta-analysis of Faris et al. (30). Chasens et al. (93) reported daytime sleepiness in older adults is negatively associated with some measures of PA. It appears practicing PA during Ramadan may explain lower ESS scores observed in the active compared to the sedentary group. Nevertheless, the mechanism(s) explaining the relationship between daytime sleepiness and PA activity level is not fully understood.

Despite the increase in ISI total scores in both groups from before Ramadan to during Ramadan, it remained clinically non-significant (i.e., total ISI score < 7), and this finding could be related to the inexistence of very high mean age in both groups (i.e., mean age < 65 years). Some previous studies suggest the rise of insomnia severity problems is associated with getting old and that about 12–25% of individuals aged more than 65 years old suffer from insomnia problems (94, 95).

The deterioration of sleep-wake patterns may lead to a subsequent decrease in cognitive function during RDIF in both groups. The digital cognitive assessment battery revealed enhancements in several cognitive domains, in the active elderly group compared to the control group. These improvements may be attributed to the effects of regular PA, combined with intermittent fasting.

Associative learning is defined as, “learning about the connection between two disparate stimuli” (96). There are not yet any studies investigating the effects of RDIF on associative learning, but we suggest the performance decrease in the control group could be attributed to the aging process, which is related to dysfunction in dopamine receptors and transmitter substances (97, 98). This may lead to the disruption in dopamine receptor stimulation levels in the prefrontal cortex, which may worsen the balance of cognitive stability and flexibility (99), consequently disturbing associative learning modulation (100). Future investigations based on clinical data regarding the dopamine pathways and their interactions with associative learning for elderly individuals are warranted. Moreover, the dysfunction of nitric oxide (NO) activity in the brain due to the aging process may also explain the learning impairment (101–103). However, regular PA reshapes the system balance, leading to the regulation of dopamine levels in the brain and the availability of more dopamine receptors (104), as well as increasing the production of NO release from endothelial cells, favoring vascular function and decreasing NO from inducible NO synthase in the brain (103, 105, 106), potentially reducing the harmful effect of aging. This is evident for active elderly who maintain the associative learning level during RDIF.

Attention refers to the collection of adapted brain functions that enable effective and adaptive behavioral selection (107). According to the BOLD-fMRI research, compensatory mechanisms exist to help attention and focus and inhibit extraneous mental processes induced by sleep deprivation, which may mitigate some of their adverse effects and add flexibility in tasks that deserve attention and vigilance (108–110). Despite the sleep deterioration observed in the sedentary group during Ramadan, attention scores increased. This may be explained by the compensatory mechanisms enabling attention improvement during Ramadan. It should be acknowledged attention scores increased in the active group, but with a higher effect size (i.e., *d* = 0.60) compared to that of the sedentary group (i.e., *d* = 0.49) and this highlights the role of continuous PA in potentially enhancing attention in the elderly (111, 112).

Furthermore, our results are in accordance with the study of Tian et al. (42), which revealed a beneficial effect of RDIF on visual attention for Muslim athletes. However, other studies revealed a significant decline in attentional performance induced by RDIF in sedentary individuals (88, 113, 114).

For executive function, which is defined as a set of mental aptitudes that incorporate working memory, adaptable reasoning, and self-control (115), physically active older adults performed better during RDIF compared to sedentary participants who did not record a change from before to during RDIF. This stagnation in the control group is in concert with the findings of Harder-Lauridsen et al. (116), who revealed no effects of intermittent fasting on executive function. Furthermore, the significant increase of executive function values for the active group reinforces existing literature suggesting regular physical exercise may buffer the impacts of aging on cognitive decrements, especially with regard to executive function (117–119). Moreover, the recent meta-analysis of Xiong et al. (120) confirmed the beneficial effects of regular physical activities for improving cognitive flexibility, working memory, and inhibitory control of executive function in cognitively healthy older adults.

Inhibitory control or inhibition refers to the ability to suppress or control impulsive (or instinctive) responses and produce replies by employing attention and logic (121). In this context, inferior inhibitory control shown in the control group, compared to physically active elderly before and during RDIF, may explain the decrease of sleep efficiency for sedentary participants (122). Ghayour Najafabadi et al. (25) showed no adverse effects of RDIF on inhibitory performance in female athletes; thereby we suggest that regular exercise may preserve inhibitory control, exhibited by faster response speeds during inhibition tasks. This is related to greater productivity of neural circuits (123), suggesting the synthesis of BDNF as a vital component underlying the impact of PA on brain structure and capacities evidently on inhibitory function (124).

Processing speed, which reflects the amount of time needed to react to and/or process information to perform an intellectual task (125), remained unchanged from before to during RDIF for both groups. Our results are contradictory to those of Tian et al. (42). This latter showed a significant effect of RDIF on psychomotor function in athletes who had poorer scores in the afternoon (i.e., at 16:00 h) than morning (i.e., at 09:00 h) (42), which could explain the reason we found no significant changes in the active group since our testing was performed in the afternoon. Additionally, higher processing speed values recorded before and during RDIF in the active group may explain the positive effects of PA training on processing speed (126). Moreover, the decrease, even though non-significant, shown in the control group between before to during RDIF, may be attributed to decreased integrity of the cerebral white matter associated to the aging process (127). Reducing this decline is important as it may cause several problems in daily life. In this context, decreases in processing speed are the strongest indicator of driving cessation in older adults (128).

To the best of our knowledge, the present study is the first to assess memory function based on eye tracking in older adults during RDIF. Novelty preference score is an operation of recognition memory based on ETM which is the capacity to identify already experienced objects (129), in addition to associative memory which is defined as the capacity to learn and retain the connection between unrelated items. Our results revealed a better increase during RDIF for active subjects compared to sedentary older adults. Similar to this unchanged value in the control group, Doniger et al. (130) found no evidence of a substantial impact of fasting (i.e., religious orthodox Jewish day of fast) on verbal recognition memory performance based on computerized cognitive assessment for normal individuals. However, a recent study conducted by Dias et al. (131) affirmed the protentional effects of intermittent fasting including several neurological modifications on memory consolidation.

Furthermore, the continuous physical training program based on several tactical schemes and combinations of team sport and repetitive movements on circuit training, as in our research, could have positively consolidated and strengthened memory functions in elder adults (132–134).

### Limitations

Despite the novelty of our study, some limitations should be acknowledged and addressed in future investigations. First, we did not evaluate sleep quality and daytime sleepiness using objective measurements (e.g., actigraphy, polysomnography, the Multiple Sleep Latency Test). Although there is disagreement about what exactly constitutes excellent sleep, sleep quality was previously described as a subjective perception (135). Interestingly, the used questionnaires include a standardized scoring strategy which seems to offer more prominent reliability and reproducibility in further investigations. The second issue is that the Neurotrack battery’s cultural adaptation did not cover all of the assessments’ components (e.g., the items on display when taking the exam). It is worth noting that for adapting cognitive assessments to different cultural contexts, there are currently no established standards guidelines, or criteria (136). Nevertheless, an exact transcribing methodology was performed to consider any potential cultural context-related performance bias. In this regard, methodological variations and the absence of sensitive computerized neuropsychological tools (i.e., the lack of objective assessment) in some studies may be to blame for the contradictory results. Third, the evident decrease in PASE scores in both groups could not be due to the decline of PA level, specifically for the active group, since coaches prioritize maintenance during RDIF to the same duration and intensity in training sessions as before Ramadan month. This energy expenditure decline may be attributed to the decrease in daily life behaviors (e.g., gardening). In this regard, raising these daily activities to normal rates is recommended. Fourth, we did not investigate hydration status, nutritional intake, and glycemia before and during Ramadan as well as in pre-dawn meal and when breaking fast, despite the existence of several studies in the elderly population confirming the strong relationship between hypohydration, nutrition and poor cognitive performance (137–142), as well as between glycemic control and cognitive functions (143, 144). More rigorous studies investigating these parameters before and during overall RDIF are needed. Other factors such as environmental variables (e.g., noise and number of the household in the same house), as well as the emotional and psychological status (e.g., satisfaction, depression), may have influenced the results. Therefore, these confounding factors should be assessed/controlled during future investigations. Furthermore, the results of our study pertain only to older adults. Muslim and could not be generalized to non-Muslim older adults given that intermittent fasting performed by our participants is of a religious type. More empirical studies with largest number of participants in non-Muslim countries should be investigated. Moreover, experimental investigations based on clinical data (e.g., biochemical and cardiometabolic parameters) are warranted to better understand the mechanisms, and objectively discuss the variation in the outcomes of interests in order to reach more definitive conclusions.

## Conclusion

Ramadan observance worsened sleep quality and increased daytime sleepiness in both groups; however, this was more prominent in the control group. Likewise, more pronounced adverse impacts on cognitive functions were recorded for inactive subjects compared to active older adults. Therefore, continuous PA practice during the month of Ramadan may counteract the potential negative effect of RDIF on sleep and cognitive performance.

## Data availability statement

The original contributions presented in this study are included in the article/supplementary material, further inquiries can be directed to the corresponding author.

## Ethics statement

The studies involving human participants were reviewed and approved by the Research Ethics Committee CPP SUD N°0322/2021 gave its approval to this project, and the present study was carried out according to the Declaration of Helsinki. The patients/participants provided their written informed consent to participate in this study.

## Author contributions

All authors listed have made a substantial, direct, and intellectual contribution to the work, and approved it for publication.
